# The HMGB1–CXCL12 Complex Promotes Inflammatory Cell Infiltration in Uveitogenic T Cell-Induced Chronic Experimental Autoimmune Uveitis

**DOI:** 10.3389/fimmu.2017.00142

**Published:** 2017-02-14

**Authors:** Juan Yun, Guomin Jiang, Yunsong Wang, Tong Xiao, Yuan Zhao, Deming Sun, Henry J. Kaplan, Hui Shao

**Affiliations:** ^1^Department of Ophthalmology and Visual Sciences, Kentucky Lions Eye Center, University of Louisville, Louisville, KY, USA; ^2^Department of Ophthalmology, Tangshan Gongren Hospital, Tangshan, China; ^3^Department of Pharmaceutical Sciences, Sullivan University College of Pharmacy, Louisville, KY, USA; ^4^Department of Ophthalmology, Doheny Eye Institute, David Geffen School of Medicine, UCLA, Los Angeles, CA, USA

**Keywords:** autoimmune disease, uveitis, immune regulation, autoreactive T cells, damage-associated molecular patterns, HMGB1, CXCL12, CXCR4

## Abstract

It is largely unknown how invading autoreactive T cells initiate the pathogenic process inside the diseased organ in organ-specific autoimmune diseases. In experimental autoimmune uveitis (EAU) induced by uveitogenic, interphotoreceptor retinoid-binding protein (IRBP)-specific T cells (tEAU) in mice, we have previously reported that high mobility group box 1 (HMGB1) released as a consequence of the direct interaction between IRBP-specific T cells and retinal parenchymal cells is an early and critical mediator in induction of intraocular inflammation. Our present study explored the roles of HMGB1 in intraocular inflammation, focusing on its role in recruiting inflammatory cells into the eye. Our results showed that supernatants from retinal explants either stimulated with HMGB1 or cocultured with IRBP-specific T cells attracted leukocytes. Notably, HMGB1 antagonists blocked supernatant-induced chemoattraction when present from the start of coculture, but not when added to the culture supernatants after coculture, indicating that molecules released by HMGB1-treated retinal cells are chemoattractive. Moreover, CXCL12 levels in the coculture supernatants were dependent on HMGB1, since they were increased in the cocultures and reduced when HMGB1 antagonists were added at the beginning of the coculture. When either anti-CXCL12 Ab was added to the supernatants after coculture or the responding lymphocytes were pretreated with Ab against CXCL12 specific receptor, CXCR4, chemoattraction by the coculture supernatants was decreased. Finally, induction of tEAU was significantly inhibited by a CXCR4 antagonist, AMD3100, at the time of autoreactive T cell transfer. Our study demonstrates that, at a very early stage of intraocular inflammation initiated by uveitogenic autoreactive T cells, synergism between HMGB1 and CXCL12 is crucial for the infiltration of inflammatory cells.

## Introduction

Autoimmune uveitis is a group of potentially visually disabling intraocular inflammatory diseases that arise without a known infectious trigger. Although the etiology remains unclear, it is generally believed that a T cell-mediated immune response to unique ocular proteins underlies the pathogenesis of the disease and this is supported by the observation that adoptive transfer of uveitogenic autoreactive T cells into susceptible, syngeneic rodents induces experimental autoimmune uveitis (tEAU) (1–4). Studies in rodent models of experimental autoimmune uveitis (EAU) induced by immunization of a well-characterized uveitogenic autoantigen, interphotoreceptor retinoid-binding protein (IRBP), have shown that activation of autoreactive T cells is a key pathogenic event linked to disease induction, progression, and recurrence (2, 4–7). While a great deal of information is available about the development and activation of autoimmune T cells in the periphery in EAU, the mechanism by which the low frequency of infiltrating uveitogenic T cells triggers and maintains the intraocular inflammatory cascade is unknown. The tEAU model, which, in contrast to the antigen-immunization EAU model, is induced without injecting microbial products and resembles human chronic uveitis, allows us to study the behavior of infiltrating effector T cells inside the eye.

Using this tEAU model, we have previously demonstrated that, once peripheral activated IRBP-specific T cells enter the eye, they interact with parenchymal cells, resulting in the production of high mobility group box 1 (HMGB1) by these cells (8). Released HMGB1 in the eye plays an early and critical role in IRBP-specific T cell-induced intraocular inflammation, because neutralization or blockade of HMGB1 using antagonists reduces ocular inflammation and suppresses uveitogenic T cell function, such as IRBP-specific T cell proliferation and cytokine production (8). We also found that HMGB1 is actively secreted within the eye within 24 h after IRBP-specific T cell transfer as a consequence of direct cell–cell contact between infiltrating IRBP-specific T cells and viable retinal cells (8), which is mediated by the Fas/FasL interaction (9). However, the mechanisms by which released HMGB1 induces intraocular inflammatory cascade are unknown. In the present study, we aimed to define the role of HMGB1 in inflammatory cell infiltration into the eye, one of the major pathogenic events initiated by the few IRBP-specific T cells that enter the eye.

High mobility group box 1 is one of the most important damage-associated molecular pattern molecules. Although itself is only a weak inflammatory mediator, its interaction with receptor for advanced glycation end products (RAGE) (10) or TLRs (11) leads to increased production and release of cytokines and other inflammatory molecules. HMGB1 also binds to nucleosomes, RNA and DNA, lipopolysaccharide, thrombospondin, triggering receptor expressed on myeloid cells-1, CD24, and CXCL12, promoting inflammatory responses (10). Three isoforms of HMGB1 have been discovered, with disulfide HMGB1 inducing cytokine production *via* TLR4, fully reduced HMGB1 promoting chemotaxis by binding CXCL12 for stimulation *via* CXCR4, and the fully oxidized HMGB1 being inactive (12).

We therefore investigated whether the interaction of autoreactive T cells and retinal cells leads to cooperation of HMGB1 and CXCL12 in promoting leukocyte migration *in vitro* and *in vivo*, and whether blocking HMGB1/CXCL12 complex inhibits its chemoattractive function, resulting in the reduction of intraocular inflammation.

## Materials and Methods

### Animals and Reagents

Eight- to ten-week-old female C57BL/6J (B6) mice, purchased from the Jackson Laboratory (Bar Harbor, ME, USA), were housed and maintained in the animal facilities of the University of Louisville (KY, USA). All animal studies conformed to the Association for Research in Vision and Ophthalmology statement about the use of animals in ophthalmic and vision research. The protocol (#14052) was approved by the Institutional Animal Care and Use Committee of the University of Louisville.

All T cells were cultured in complete medium [RPMI 1640 medium (Mediatech, Manassas, VA, USA) supplemented with 10% fetal calf serum (Hyclone, Logan, UT, USA), 5 × 10^−5^ M 2-mercapatoethanol, and 100 µg/ml of penicillin/streptomycin]. The human IRBP peptide 1–20 (GPTHLFQPSLVLDMAKVLLD) was synthesized by Sigma-Aldrich (St. Louis, MO, USA). The fully reduced HMGB1 (Cat#HM-115) was purchased from HMGBiotech (Milano, Italy) and chicken anti-HMGB1 polyclonal antibody (Cat#326052233) from Shino-Test Corporation (Kanagawa, Japan).

### Induction of tEAU

The method used to induce tEAU has been reported previously (4). Briefly, T cells from mice immunized 12 days previously with peptide IRBP_1–20_ were purified from draining lymph nodes and spleen cells by passage through a nylon wool column (Polysciences, Warrington, PA, USA), then 1 × 10^7^ cells in 2 ml of RPMI 1640 medium were added to each well of a six-well plate (Costar, Corning, NY, USA) and stimulated with 20 µg/ml of IRBP_1–20_ in the presence of 1 × 10^7^-irradiated syngeneic spleen cells as antigen-presenting cells (APCs). After 2 days, activated lymph blasts were isolated by gradient centrifugation on Lymphoprep (Sigma-Aldrich) and injected intraperitoneally (i.p.) in 0.2 ml of PBS into naive B6 recipients (5 × 10^6^ cells/mouse). The clinical course of the disease was assessed by indirect fundoscopy once or twice a week and graded as described previously (13).

### Pathological Examination

Inflammation of the eye was confirmed by histopathology. Whole eyes were collected, immersed for 1 h in 4% phosphate-buffered glutaraldehyde, and transferred to 10% phosphate-buffered formaldehyde until processed. The fixed and dehydrated tissue was embedded in methacrylate, and 5 µm sections were cut through the pupillary-optic nerve plane and stained with hematoxylin and eosin. Presence or absence of disease was evaluated blind by examining six sections cut at different levels for each eye. Severity of EAU was scored on a scale of 0 (no disease) to 4 (maximum disease) in half-point increments based on the presence of inflammatory cell infiltration of the iris, ciliary body, anterior chamber (AC), and retina (4).

### Isolation of Retinal Explants and Coculture with Activated IRBP_1–20_-Specific T Cells or HMGB1

Eyes were collected from B6 mice and neural retinas isolated and used as retinal explants as described previously (8, 9). Retinal explants were placed with the inner membrane facing up in a 24-well plate and cultured in 500 µl of DMEM/F12 medium (Mediatech Inc., Manassas, VA, USA) containing 0.1% fetal calf serum, then either HMGB1 (0.1 or 1 µg/ml) or 5 × 10^4^-activated T cells, prepared from IRBP_1–20_-immunized mice at day 11–14 postinjection as described above, was added and the cells incubated at 37°C with 5% CO_2_ for 6 h, then CXCL12 and HMGB1 levels in the supernatants were measured by ELISA, as described below. In inhibitor studies, retinal explants were cocultured with activated IRBP_1–20_-specific T cells in the presence or absence of anti-HMGB1 mAb (IBL international GmbH, Toronto, ON, Canada), anti-RAGE mAb (R&D, Minneapolis, MN, USA), or glycyrrhizin (Calbiochem) for 6 h, then the supernatants were collected for ELISA or chemotactic assays.

### Intraocular Inoculations

B6 mice were anesthetized by i.p. injection of ketamine (80 mg/kg, JHP Pharmaceuticals, Rochester, MI, USA) and xylazine (10 mg/kg, Akorn, Decatur, IL, USA). One drop of 0.5% tropicamide and 1.25% phenylephrine hydrochloride ophthalmic solutions was applied topically on the eye before injection. Under a dissecting microscope, 1 µl of 1 µg anti-HMGB1 Ab was injected into the AC, one eye with a microliter syringe and a 33-gauge needle (Hamilton, Reno, NV, USA).

### Intraocular Fluid Collection

The removed eyeball was immersed in 200 µl of PBS and cut in two, then the cornea, sclera, and lens were discarded and the rest of the tissue cut into small pieces and the suspension containing the aqueous humor, vitreous fluid, and fine pieces of tissue centrifuged at 500 *g* for 5 min at 4°C, then the supernatant (intraocular fluid) was immediately stored in a −80°C freezer until use. Half (about 100 µl) of each collection from one eyeball was used for CXCL12 measurement by ELISA.

### Isolation of Eye-Infiltrating Cells

Eyes were collected after PBS perfusion, and a cell suspension was prepared by digestion for 10 min at 37°C with collagenase (1 mg/ml) and DNase (100 µg/ml) in RPMI 1640 containing 10% FCS. The cells were washed, re-suspended in staining buffer (PBS containing 3% FCS and 0.1% sodium azide), and stained with fluorescent mAbs to identify inflammatory cells by flow cytometry.

### ELISA for HMGB1 and CXCL12

Culture supernatants from retina explants or intraocular fluid, prepared as described above, were added to wells pre-coated with HMGB1 (Abcam, Cambridge, MA, USA) or CXCL12 capture Abs (R&D System, Minneapolis, MN, USA) and levels of HMGB1 or CXCL12 measured following the manufacturer’s instruction.

### Immunohistochemistry for CXCL12 and CXCR4

To detect expression of CXCL12 and CXCR4 on the retina, paraffin-embedded tissue slides were deparaffinized and rehydrated with xylene and 100, 95, and 80% ethanol. After antigen retrieval in a citrate-buffered solution in a boiling water bath, the tissue was blocked by incubation with 3% BSA for 1 h at room temperature, then the slides were double-stained by overnight incubation at 4°C with phycoerythrin (PE)-labeled anti-CXCL12 Ab (R&D) or anti-CXCR4 Ab (R&D) and fluorescein isothiocyanate (FITC)-labeled anti-glutamine synthetase (GS) Ab (Sigma, St. Louis, MO, USA) or anti-Iba-1 Ab (Abcam, Cambridge, MA, USA), then the nuclei were counterstained with DAPI (Sigma) and the slides examined by fluorescence microscopy.

### *In Vivo* Treatment with AMD3100, a Specific Inhibitor of CXCR4

We followed the protocol for AMD3100 treatment previously described by Matthys et al. (14). The mice were anesthetized by i.p. injection of ketamine (80 mg/kg) and xylazine (10 mg/kg), and a number 2002 Alzet osmotic minipump (Alza, Palo Alto, CA, USA) was implanted dorsolaterally under the skin. The pumps were filled with 5 mg of AMD3100 in 90 µl of PBS, which was delivered at a rate of 0.25 µl/h (357 µg/day) for 14 days. Groups of mice implanted with pumps containing only PBS were also included. Other untreated mice were anesthetized like the treated ones but were not implanted with pumps.

### Assays for IRBP-Specific T Cell Proliferation and Cytokine Production

Nylon wool-enriched T cells prepared at 15 days after transfer of IRBP_1–20_-specific T cells into B6 mice were seeded at 4 × 10^5^ cells/well in 96-well plates and cultured at 37°C for 60 h in 200 µl of complete medium with or without the indicated concentration of IRBP_1–20_ in the presence of irradiated syngeneic spleen APCs (1 × 10^5^), and [^3^H]thymidine incorporation during the last 8 h assessed using a microplate scintillation counter (Packard Instruments). The proliferative response was expressed as the mean cpm ± SD for triplicate samples or the proliferative stimulus index, calculated as the mean cpm for antigen-stimulated cultures/mean cpm for unstimulated controls ratio, for triplicate samples. To measure cytokine production by responder T cells, supernatants were collected 48 h after T cell stimulation and assayed for IFN-γ, IL-17, and IL-10 using ELISA kits (R&D).

### Chemotaxis Assay

Splenocytes (3 × 10^5^ cells/well) from naïve B6 mice that had either been left untreated or been incubated for 30 min with 50 µg/ml of anti-CXCR4 Ab, then washed, were added to the upper wells of 24-well Transwell micro-chemotaxis devices (5 µm pore size; Costar) and either medium with or without HMGB1 or CCL2 or supernatants from cell cultures or cocultures was added to the lower wells. In inhibitor studies, the inhibitor was added either at the beginning of cocultures or to the coculture supernatant at the end of coculture. After 2 h, cells that had migrated to the lower wells were collected, counted, stained with antibodies against CD3 for T cells, CD11b for macrophages, or Gr-1 for granulocytes, and examined by flow cytometry. The chemotactic index was calculated as the ratio of the number of migrated cells in chemoattractant-containing wells divided by the number of migrated cells in medium-containing wells. All assays were performed in triplicate.

### Statistical Analysis

Experiments were repeated at least three times. An unpaired Student’s *t*-test for two sets of data, one-way or two-way ANOVA for three or more means, or the Mann–Whitney *U* test for the pathological score of uveitis was used for statistical analysis. A *p* value < 0.05 was considered significant.

## Results

### HMGB1, Released as a Consequence of the Interaction between Uveitogenic T Cells and Retinal Cells, Is Critical for the Production of Chemotactic Molecules by Retinal Cells

**Figure 1:**
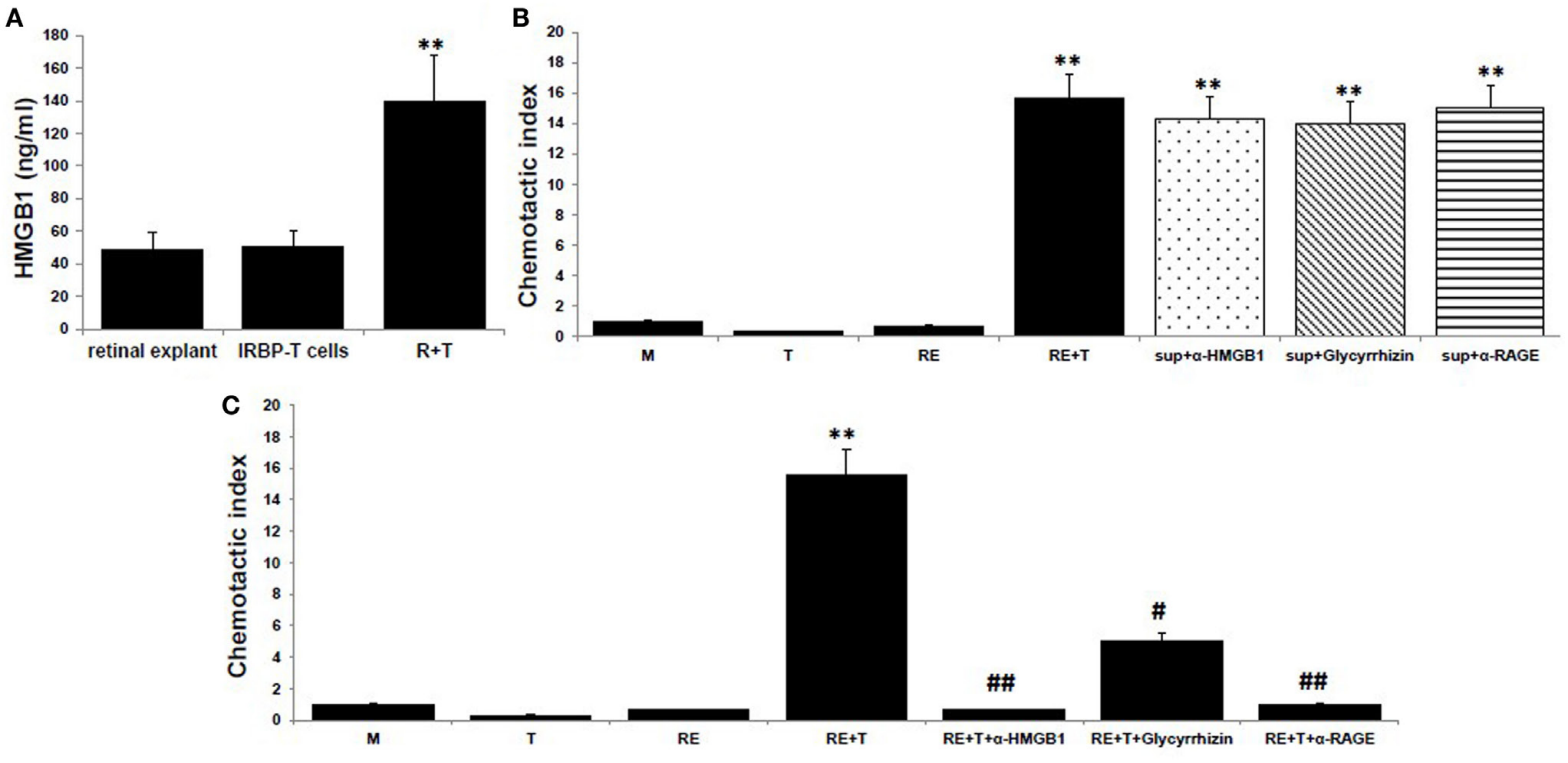
**High mobility group box 1 (HMGB1) release as a consequence of the interaction of uveitogenic T cells and retinal cells is critical for the production of chemotactic molecules by retinal cells**. **(A)** Retinal explants from naïve B6 mice or activated interphotoreceptor retinoid-binding protein (IRBP)-specific T cells were cultured alone or together for 6 h, then culture supernatants were assayed for HMGB1 by ELISA. **(B,C)** Chemotactic index of medium or the supernatant from the T cells or retinal explants or the supernatant from the cocultures either alone or with anti-HMGB1 Ab (8 µg/ml), glycyrrhizin (200 µg/ml), or anti-receptor for advanced glycation end products (RAGE) Ab (30 µg/ml) added at the end of the coculture **(B)**, or at the beginning of the coculture **(C)**. The results are representative of those for three experiments. **p < 0.01 compared to medium alone, ^#^*p* < 0.05 and ^##^*p* < 0.01 compared to the coculture of retinal explants and IRBP-specific T cells in one-way ANOVA.

To examine the chemotactic ability of HMGB1 released after the interaction between uveitogenic T cells and retinal cells, we collected the supernatants from cocultures of activated IRBP-specific T cells and retinal explants for an *in vitro* chemoattractant migration assay. In this assay, splenocytes from naïve B6 mice were added to the top well of a chemotaxis chamber and coculture supernatants alone or together with 8 µg/ml of anti-HMGB1 mAb, 200 µg/ml of glycyrrhizin (an HMGB1 inhibitor), or 30 µg/ml of anti-RAGE mAb was added to the lower well, then the number of cells that migrated to the lower well after 2 h was counted by flow cytometry. As shown in Figure 1A, HMGB1 was released into the medium of the cocultures, as shown previously in Ref. (8). These supernatants were chemoattractive for splenocytes, both alone and after addition of anti-HMGB1 Ab, glycyrrhizin, or anti-RAGE Ab (Figure 1B). However, when the same concentration of anti-HMGB1 Ab, glycyrrhizin, or anti-RAGE Ab was added at the beginning of the coculture of retinal explants and activated IRBP-specific T cells, the supernatants from these cocultures no longer attracted splenocytes (Figure 1C), showing that HMGB1 and RAGE are involved in the release of a chemotactic molecule produced during the interaction between T cells and retinal cells.

### HMGB1 Alone Cannot Recruit Inflammatory Cells, Whereas Supernatants from Retinal Explants Stimulated with HMGB1 Do Recruit Inflammatory Cells

**Figure 2:**
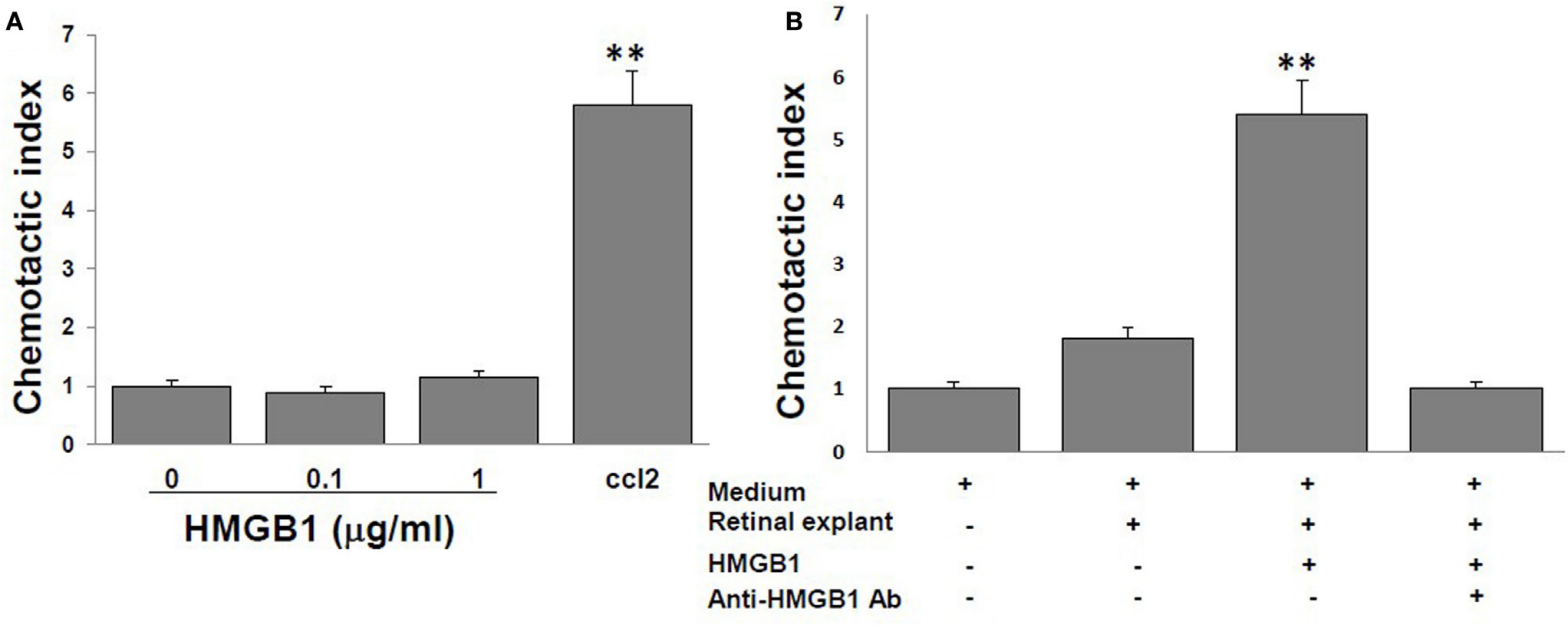
**High mobility group box 1 (HMGB1) alone cannot induce splenocyte migration, whereas supernatants collected from retinal explants stimulated with HMGB1 can**. Splenocytes from naïve B6 mice were added to the top well of the chemotaxis chamber. **(A)** The chemotactic index of different concentrations of HMGB1 or 10 nM CCL21. **(B)** The chemotactic index of the medium only or supernatant from cultures of retinal explants incubated for 6 h with medium or 1 µg/ml of HMGB1 in the presence or absence of 1 µg/ml of anti-HMGB1Ab. **p < 0.01 compared to medium alone in one-way ANOVA.

High mobility group box 1 alone is reported to have no chemotactic activity to leukocytes (15, 16), and we confirmed this in Figure 2A, in which no migration of splenocytes from naïve B6 mice was seen in the medium containing 0, 0.1, or 1 µg/ml of reduced form of HMGB1, contrast to that of the medium containing 10 nM CCL12, a chemoattractant for lymphocytes. However, as shown in Figure 2B, supernatants collected from retinal explants stimulated with 1 µg/ml of HMGB1 did attract splenocytes, while supernatants from the same cells incubated with HMGB1 and 1 µg/ml of anti-HMGB1 Ab did not. The results show that molecules released from HMGB1-stimulated retinal cells attract immune cells.

### CXCL12 Is the Molecule Induced by HMGB1 That Attracts Immune Cells

**Figure 3:**
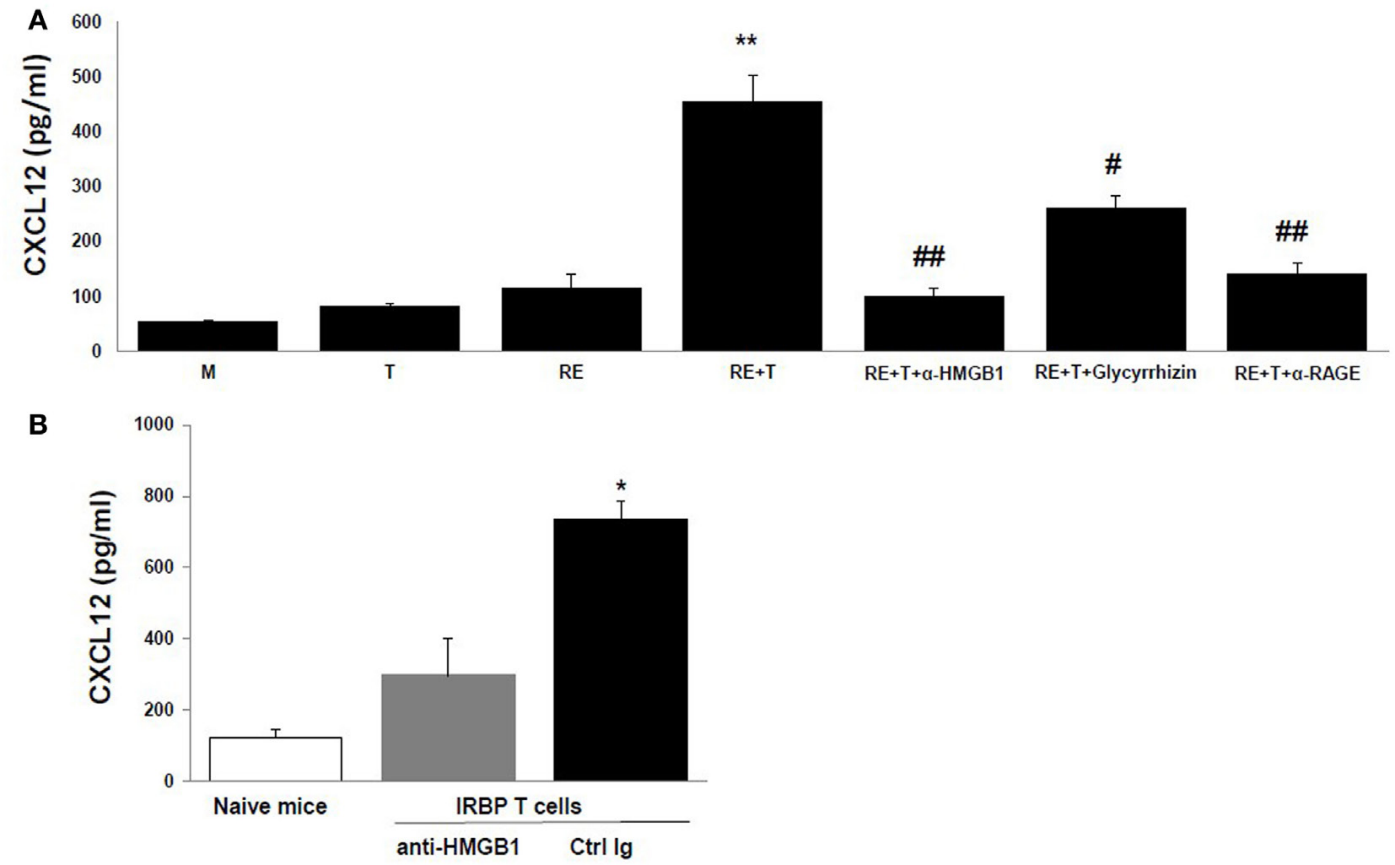
**High mobility group box 1 (HMGB1) induces retinal cells to secrete CXCL12**. **(A)** Medium or the supernatant from the T cells or retinal explants or the supernatant from the cocultures alone or with addition of anti-HMGB1 Ab, glycyrrhizin, or anti-receptor for advanced glycation end products (RAGE) Ab at the same doses as Figure 1B at the beginning of the coculture was assayed for CXCL12 by ELISA. **p < 0.01 compared to medium alone, ^#^*p* < 0.05 and ^##^*p* < 0.01 compared to the coculture of retinal explants and IRBP-specific T cells in one-way ANOVA. **(B)** Naïve B6 mice were left untreated or were adoptively transferred with activated IRBP_1–20_-specific T cells (5 × 10^6^ cells/mouse) and 1 µg of either anti-HMGB1 Ab or control Ig was injected into the anterior chamber of each eye. Next day, the intraocular fluid was collected and CXCL12 levels measured. The data are the mean ± SD for eight eyes per group. *p < 0.05 compared to the anti-HMGB1 Ab-treated eye in one-way ANOVA.

The HMGB1-induced recruitment of inflammatory cells depends on CXCL12, and HMGB1 and CXCL12 form a heterocomplex that binds exclusively to CXCR4 (15–17). We therefore speculated that the chemotactic molecule released by retinal cells in the presence of HMGB1 was CXCL12. To test this, we first measured CXCL12 levels in the supernatants of retinal cells cultured alone or cocultured with IRBP-specific T cells in the presence or absence of anti-HMGB1 Ab, glycyrrhizin, or anti-RAGE Ab. As shown in Figure 3A, high levels of CXCL12 were found in supernatants from cocultures of IRBP-specific T cells and retinal explants, whereas only low amounts were detected in cultures of retinal explants or IRBP-specific T cells alone or in cocultures of retinal explants and IRBP-specific T cells in the presence of anti-HMGB1 Ab, glycyrrhizin, or anti-RAGE Ab, showing that both cell types were required and that these HMGB1 inhibitors either inhibited or reduced CXCL12 production by cocultured retinal cells. The *in vitro* increase in CXCL12 levels in cocultures of retinal explants and activated IRBP-specific T cells was confirmed *in vivo* in a study in which the transfer of IRBP-specific T cells to naïve mice was accompanied by AC injection of either anti-HMGB1 Ab or control Ig into each eye, then CXCL12 levels in the intraocular fluid were measured the next day. As shown in Figure 3B, high CXCL12 level was seen in mice that received IRBP-specific T cells and control Ig, but not anti-HMGB1 Ab.

**Figure 4:**
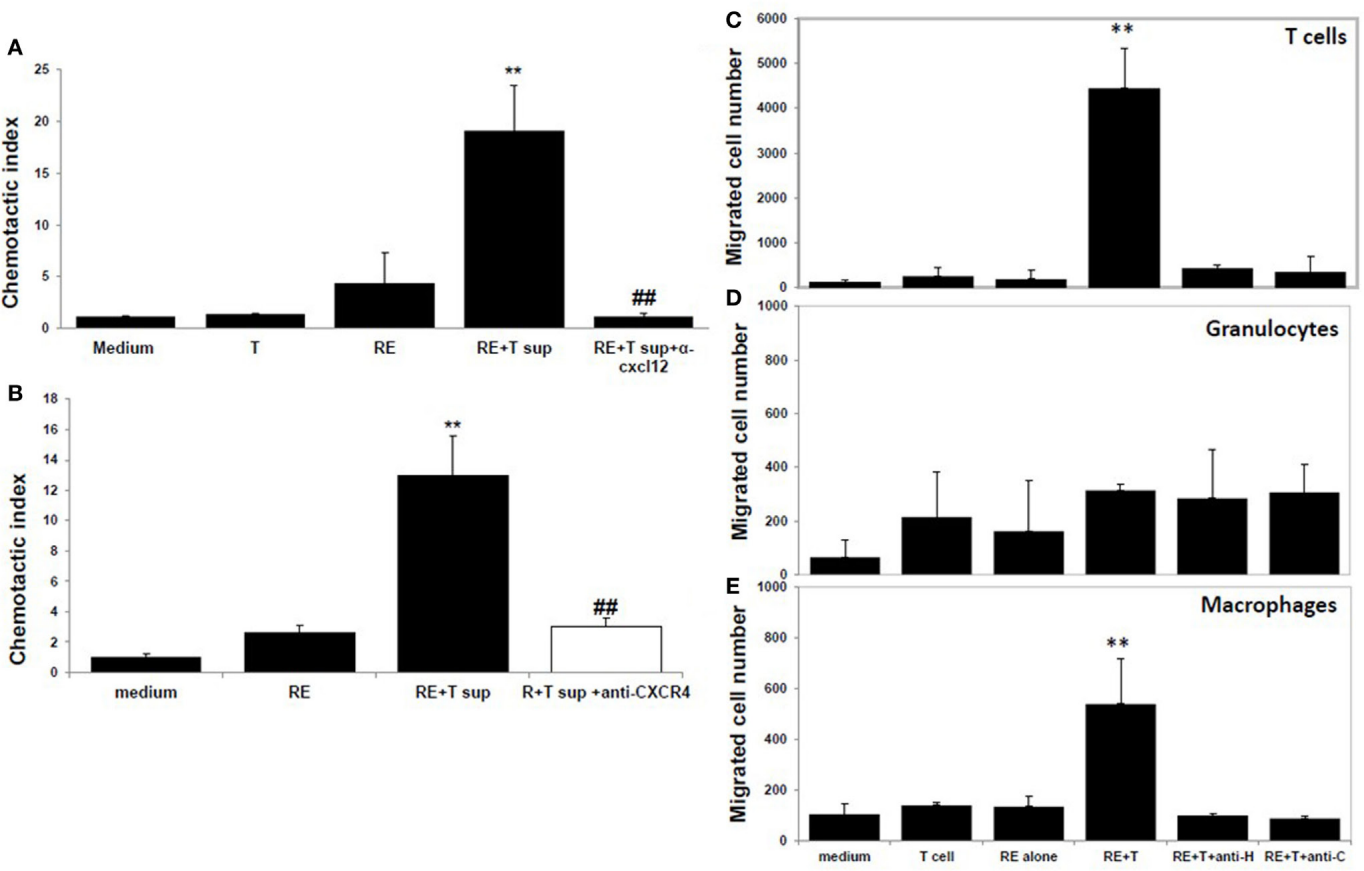
**CXCL12 is the high mobility group box 1 (HMGB1)-induced molecule that attracts T cells**. **(A)** Chemotactic index of medium or the supernatant from the T cells or retinal explants or the supernatant from the cocultures either alone or with addition of anti-CXCL12 Ab (5 µg/ml) at the end of the coculture. **(B)** Splenocytes were incubated with or without anti-CXCR4 Ab (50 µg/ml) for 30 min, then were washed and used in the migration assay as in panel **(A)**. **p < 0.01 compared to medium alone; and ^##^*p* < 0.01 compared to the coculture supernatants of retinal explants and interphotoreceptor retinoid-binding protein-specific T cells, in one-way ANOVA. **(C–E)** Medium or the supernatant from the T cells or retinal explants or the supernatant from cocultures in the presence or absence of 1 µg/ml of anti-HMGB1 (anti-H) for 6 h or supernatant from cocultures to which anti-CXCL12 Ab (anti-C; 5 µg/ml) was added after coculture was used in the migration assay and the cells that migrated into the lower chamber were stained with anti-CD3 Ab **(C)**, Ly6G Ab **(D)**, or CD11b Ab **(E)** and analyzed by flow cytometry. **p < 0.01 compared to medium alone in one-way ANOVA.

Moreover, neutralization of CXCL12 in the coculture supernatants by anti-CXCL12 Ab (Figure 4A) or prior blockade of CXCR4, the CXCL12 receptor, on the responder splenocytes using anti-CXCR4 Ab (Figure 4B) inhibited induction of splenocyte migration by coculture supernatants.

As shown by flow cytometry in Figures 4C–E, coculture supernatants from IRBP-specific T cells and retinal explants were effective in attracting both CD3^+^ T cells (Figure 4C) and CD11b^+^ macrophages (Figure 4E), and this effect was blocked by the presence of anti-HMGB1 Ab (anti-H) during the coculture of CXCL12 release or by addition of anti-CXCL12 Ab (anti-C) to the coculture supernatants. Coculture supernatants from IRBP-specific T cells and retinal explants were not effective in attracting Gr-1-positive granulocytes (Figure 4D).

### tEAU Was Attenuated by Blockade of CXCR4 Using AMD3100

**Figure 5:**
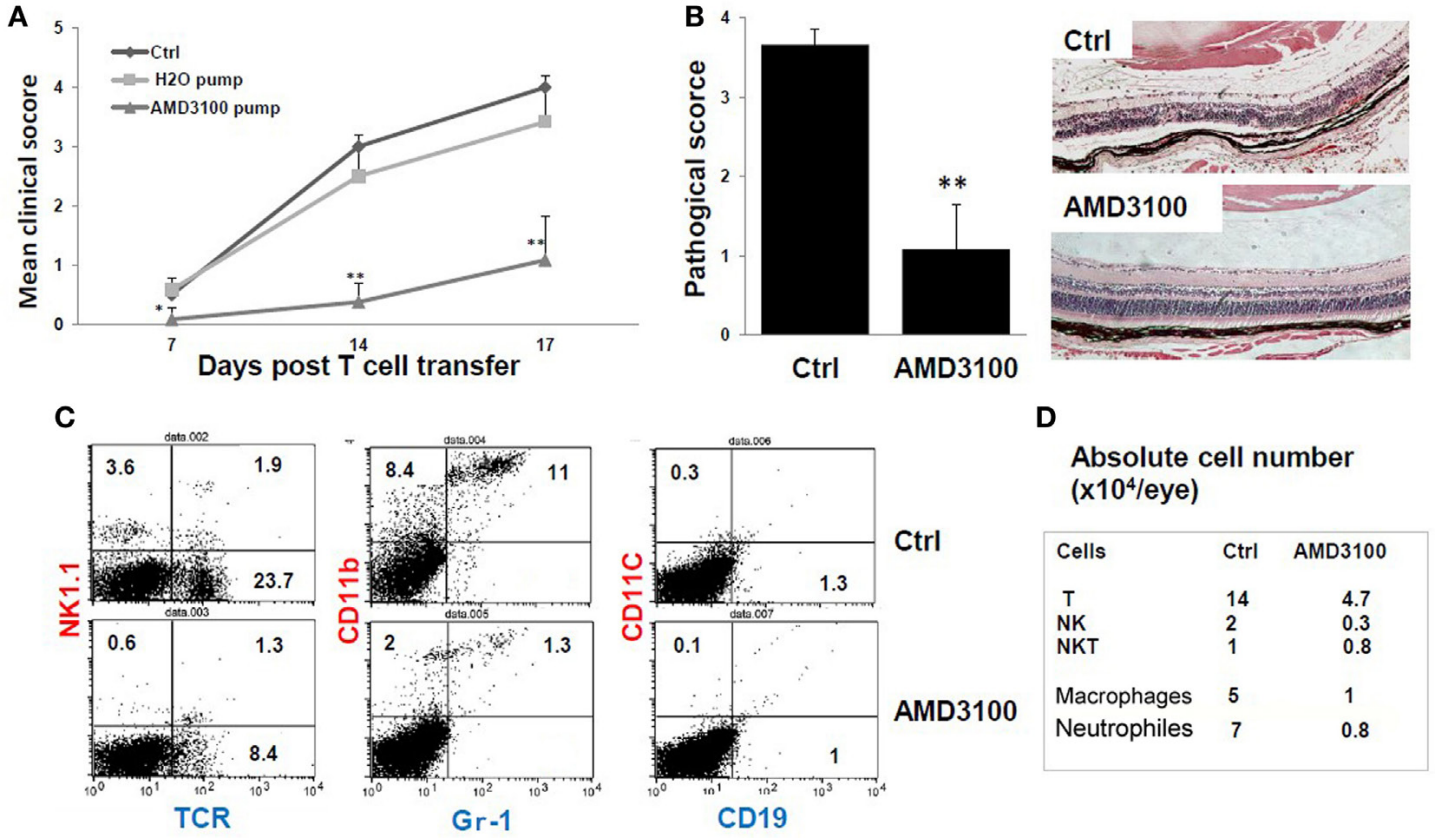
**The CXCR4 inhibitor AMD3100 significantly inhibits tEAU**. Groups of mice were implanted with pumps filled with 5 mg of AMD3100 in 90 µl of PBS (AMD3100 pump) or PBS alone (PBS pump) 1 day before interphotoreceptor retinoid-binding protein (IRBP)-specific T cell transfer as described in Section “Materials and Methods” and examined weekly for clinical score **(A)** or on day 15 for **(B)**. A group of mice was left untreated as positive controls (Ctrl). **(A)** The mean clinical score for all three groups and **(B)** the pathological score for the indicated groups (*n* = 12 mice) presented as the mean ± SE. Hematoxylin and eosin; original magnification, ×100. *p < 0.05 and **p < 0.01 compared to the Ctrl group using Mann–Whitney *U* test. **(C)** Identification of infiltrating leukocytes in the eye of day 8 post-IRBP_1–20_-specific T cell-injected mice treated with pumps containing AMD3100 or PBS (Ctrl group). A suspension of single ocular cells for each group was pooled (total five mice in control group, and four in treated group), stained with the indicated fluorescein isothiocyanate- and phycoerythrin-conjugated antibodies and analyzed by flow cytometry. The percentage of positive cells is indicated. **(D)** Summary of infiltrative leukocyte subsets, from each eye is shown. Cells recovered from eyes of each group were counted after trypan blue staining. The number of cells was calculated based on the percentage of subsets as determined by flow cytometry.

We then examined the role of HMGB1/CXCL12 *in vivo* in tEAU by comparing severity in mice with and without treatment with the CXCR4 inhibitor, AMD3100. B6 mice were implanted with osmotic minipumps releasing AMD3100 at a rate of 357 μg/day for 14 days (see Materials and Methods), a treatment regime used in a study in which AMD3100 successfully inhibited induction of collagen-induced arthritis in mice (14). Two groups of control mice were included, one implanted with pumps containing PBS (PBS pump) and one with no implant (Ctrl). One day later, all mice were injected with IRBP-specific T cells to induce tEAU, then were examined for clinical signs of intraocular inflammation. Compared with either set of controls, the AMD3100-treated mice developed much milder ocular inflammation, as determined by the clinical score (Figure 5A). Histological evaluation on day 15 post-transfer of the control group and the AMD3100 pump group showed a well preserved retinal structure and minimal vitreous infiltrate in the AMD3100-treated mice (Figure 5B).

To determine which leukocyte subsets were dominant in tEAU and blocked by AMD3100, we analyzed eye-infiltrating cells in control- and AMD3100-treated mice on day 8 after disease induction by staining pooled single eye cells from each group with a panel of antibodies specific for different leukocytes and flow cytometric analysis. As shown in Figures 5C,D, AMD3100-treated mice showed a profound reduction in infiltrating cells. The percentage of αβ T cells dropped from 23.7% in the disease controls to 8.4% in mice treated with AMD3100, and the percentage of CD11b^+^Gr-1^−^ monocytes/macrophages decreased from 8.4 to 2%. In addition, the percentage of CD11b^+^ Gr-1^+^ neutrophils was dramatically reduced (11 versus 1.3%) (Figure 5C). Moreover, the absolute number of infiltrating cells in each eye of AMD3100-treated mice was significantly reduced compared to the untreated control eye (Figure 5D). At the day 8 post-T cell transfer, there were not many infiltrating CD11C^+^ dendritic cells and CD19^+^ B cells detected in both control and treated eyes.

**Figure 6:**
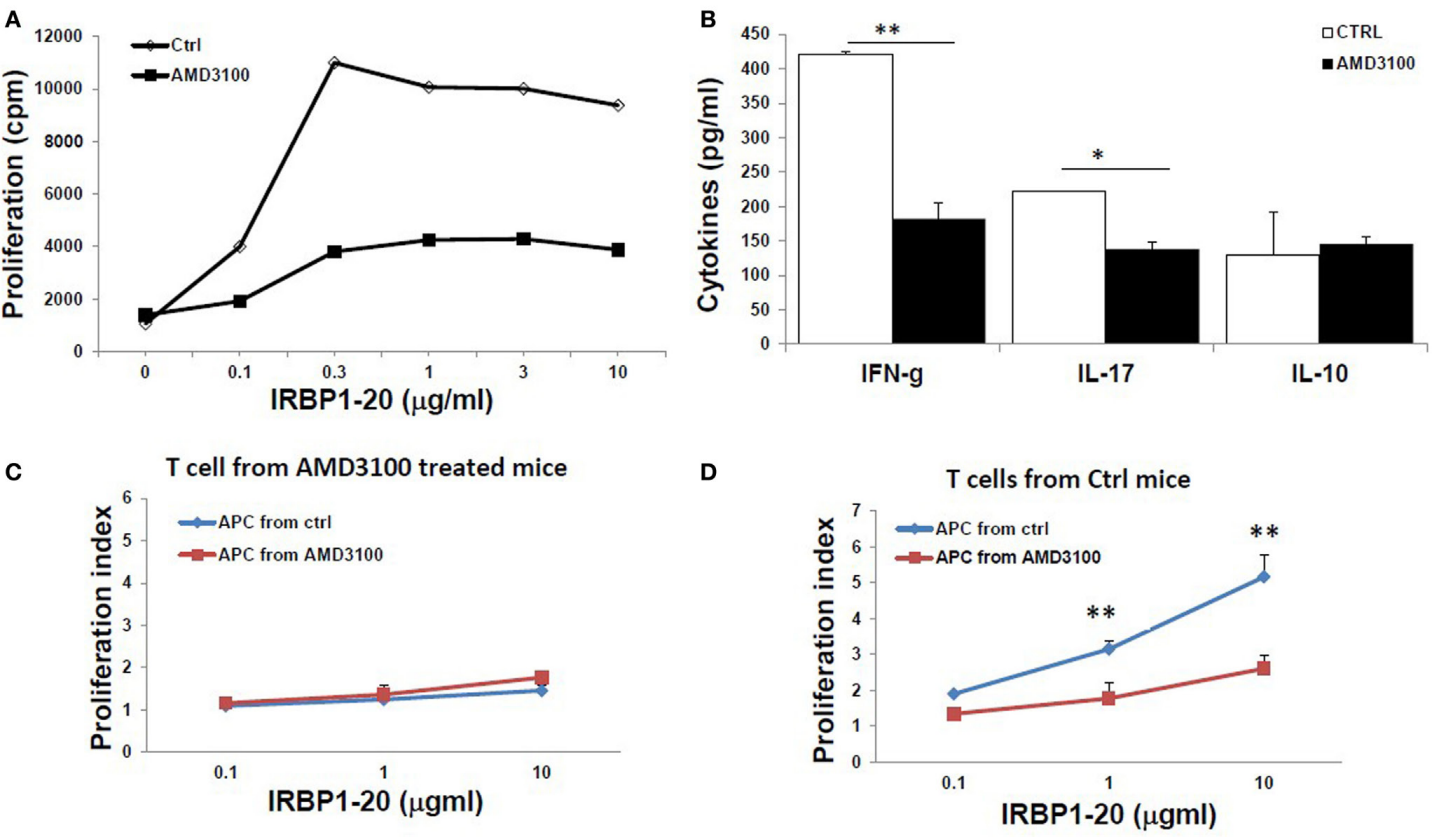
**Reduced responses of interphotoreceptor retinoid-binding protein (IRBP)-specific T cells in AMD3100-treated mice**. **(A,B)** T cells from tEAU mice treated with AMD3100 or the Ctrl group were collected on day 15 after T cell transfer and cultured with irradiated antigen-presenting cells (APCs) and increasing doses of IRBP_1–20_, then proliferation of responding T cells **(A)**, and levels of IFN-γ, IL-17, and IL-10 **(B)** released into the culture supernatants were measured. **(C,D)** Responder T cells prepared at day 8 post-IRBP_1–20_-specific T cell-injected mice treated with AMD3100 [**(C)**, five mice] or control groups [**(D)**, four mice] were incubated with increasing doses of IRBP_1–20_ in the presence of irradiated splenic APCs from either AMD3100- or control-treated mice and their proliferation measured. *p < 0.05 and **p < 0.01 compared to the Crtl group in Student’s *t*-test.

Furthermore, IRBP-specific responses of T cells were significantly reduced after treatment with AMD3100. Significant lower IRBP-specific T cell proliferation (Figure 6A) and IFN-γ and IL-17 release (Figure 6B) in response to stimulation with IRBP_1–20_ were seen with T cells from mice treated with AMD3100. However, no significant difference in IL-10 release was seen between AMD3100-treated and control mice, as we previously reported using anti-HMGB1Ab injection (8). To examine the reduced proliferation of lymphocytes a direct effect of CXCR4 inhibition or an indirect effect on antigen-presenting cells, we performed cross tests in which T cell proliferation was measured using all four combinations of responder T cells and APCs from IRBP_1–20_-specific T cell transferred and AMD3100-treated or disease control mice. T cells from AMD3100-treated mice did not respond to increasing doses of IRBP_1–20_ Ag in the presence of APCs from either AMD3100-treated or disease control mice (Figure 6C), whereas T cells from control mice reacted well in the presence of APCs from control mice, but not AMD3100-treated mice (Figure 6D), indicating that dysfunction of both T cells and APCs contributed to the T cell hyporesponsiveness in AMD3100-treated mice.

### AMD3100 Inhibits the Increase in CXCL12 and CXCR4 Expression in the Eye in tEAU

**Figure 7:**
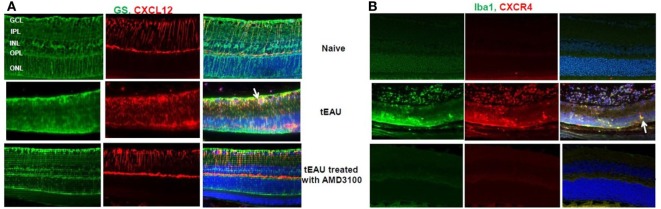
**CXCL12 and CXCR4 expression in retinas**. Expression of CXCL12 **(A)** and CXCR4 **(B)** was examined on paraffin-embedded sections of retina from naïve mice and from mice on day 15 after interphotoreceptor retinoid-binding protein-specific T cell transfer treated with or without AMD3100 by staining with phycoerythrin-conjugated anti-CXCL12 Ab [red in panel **(A)**] or anti-CXCR4 Ab [red in panel **(B)**] and fluorescein isothiocyanate-conjugated anti-glutamine synthetase (GS) Ab [green in panel **(A)**] or anti-Iba-1 Ab [green in panel **(B)**]. The right columns show the fused image. Cell nuclei are stained blue with DAPI.

Having demonstrated CXCL12 release by retinal cells after stimulation with HMGB1 or activated IRBP-specific T cells *in vitro* (Figure 3), we examined *in vivo* CXCL12 expression in naïve mice and in mice on day 15 after transfer of IRBP-specific T cells with or without AMD3100 treatment. As shown in Figure 7A (left), immunochemical staining for CXCL12 (red stain, first row, center panel) showed that CXCL12 was constitutively expressed on the retina, especially in the ganglion cell layer (GCL), inner plexiform layer (IPL), and outer plexiform layer (OPL). At the peak of disease (d15 post-T cell transfer; middle row), the retina became thinner and CXCL12 expression was increased in the neuroretina in all regions from the inner nuclear layer to the outer boundary of the outer nuclear layer. However, tEAU mice treated with AMD3100 (bottom row) showed similar CXCL12 expression to naïve mice (first row). Staining for astroglial cells using the marker GS (green stain) showed that not many GS cells expressed CXCL12 in naïve mice (first row, right panel), while during inflammation, more GS cells expressed CXCL12 (indicated by arrows, middle row, right panel), which was markedly reduced by AMD3100 treatment (bottom row, right panel). Our results suggest that CXCL12 may play a role in the maintenance of retinal cells in specific layers in the adult retina.

We also examined expression of the specific CXCL12 receptor, CXCR4, in the eye before, or after, 15 days of IRBP-specific T cell transfer with or without AMD3100 treatment. As seen in Figure 7B, no CXCR4 signal (red stain) was seen before tEAU induction (first row, center panel) indicating no endogenous expression of CXCR4 in the eye of naïve mice. However, CXCR4 was detected in the infiltrating cells at the peak of disease (middle row, center panel) and significantly reduced by AMD3100 treatment (bottom row, center panel). Staining for ionized calcium-binding molecule 1 (IBa1-1), a marker for macrophages/microglia, was markedly increased at the peak of tEAU (green, middle row, left panel) and some Iba-1-expressing cells also expressed CXCR4 (indicated by arrows, middle row, right panel), and both effects were abolished in the AMD3100-treated mice (bottom row).

## Discussion

We have previously reported that HMGB1 is detected in culture supernatants as early as 2 h of coculture of retinal explants and activated IRBP-specific T cells and at 1 day after adoptive transfer of activated IRBP-specific T cells, and blockade of HMGB1 using antagonists reduces ocular inflammation and suppresses uveitogenic T cell function (8, 9). However, how HMGB1 is involved in the pathogenesis of effector phase of uveitis is not clear.

It was recently demonstrated that HMGB1 is an enhancer of the activity of CXCL12 in stimulating the migration of mouse embryonic fibroblasts (15, 16). Our study further supports this and explores the synergistic roles of HMGB1 and CXCL12 in the infiltration of inflammatory cells at a very early stage of intraocular inflammation induced by uveitogenic autoreactive T cells. Our results in Figures 1 and 3 show that interaction of uveitogenic T cells with retinal cells initiated release of HMGB1 and CXCL12 by retinal cells. CXCL12 release was dependent on HMGB1, since CXCL12 was not released from retinal cells in the presence of an anti-HMGB1 Ab (Figure 3). This process is very important for the subsequent role of inflammatory migration. Once the complex forms, the migration-stimulating activity of the supernatant was not affected by neutralization of HMGB1 by anti-HMGB1 Ab or glycyrrhizin (Figure 1B), a glycoconjugated triterpene produced by the licorice plant *Glycyrrhiza glabra*, which has been reported to inhibit the chemoattractant and mitogenic activities of HMGB1 on 3T3 fibroblasts and binds to both HMG box domains (BoxA and BoxB) (18). We previously showed that injection of mice with HMGB1 antagonists on the same day as IRBP-specific T cell transfer inhibits uveitogenic T cell-induced intraocular inflammation, but not when given later, indicating that HMGB1-induced CXCL12 release might be an early event initiated by infiltrating effector T cells (8).

It is not known how these two molecules are structurally associated. Nuclear magnetic resonance chemical-shift mapping indicates contacts between CXCL12 and full-length HMGB1 and its individual HMG boxes (17). HMGB1 binds to CXCL12 monomers or promote binding of CXCL12 to CXCR4 by fixing the N-terminal domain of CXCL12 in the best conformation for triggering CXCR4 signaling (16). Fluorescence resonance energy transfer studies with tagged CXCR4 have indicated that the conformation of CXCR4 dimers interacting with the HMGB1/CXCL12 heterocomplex is different from the conformation of those interacting with CXCL12 alone (17).

Receptor for advanced glycation end products has been shown to be the receptor responsible for HMGB1-induced migration (19). Our study showed that RAGE was required for HMGB1-induced CXCL12 release from retinal cells and probably for the formation of the HMGB1/CXCL12 complex, but not for inflammatory cell migration *per se*, since HMGB1-induced CXCL12 release was inhibited by anti-RAGE mAb (Figure 1C), whereas HMGB1/CXCL12-induced migration of splenocytes was not (Figure 1B). The reason why a previous study found that HMGB1-induced chemotaxis of rat smooth muscle cells (SMCs) was inhibited not only by anti-HMGB1 Abs but also by anti-RAGE Abs (20), might be that anti-RAGE Abs inhibit HMGB1-induced CXCL12 release from SMCs. Our results are also compatible with those reported by Schiraldi et al. (17), who showed that, *in vitro*, CXCL12 expression and release were increased in HMGB1-stimulated mouse fibroblasts from wild-type B6 mice, but not from RAGE-deficient (RAGE^−/−^) mice, whereas migration of bone marrow cells induced by a combination of HMGB1 and CXCL12 was similar in both sets of mice, while, *in vivo*, absence of RAGE in C57BL/6 mice did not prevent, but actually promoted, recruitment of monocytes into the injured muscle, excluding the possibility that inflammatory cells were recruited through RAGE signaling. They argued that the role of RAGE in cell migration is to trigger CXCL12 transcription (21) so that CXCL12 production is increased and sustained over time and that RAGE is no longer needed when sufficient CXCL12 is produced or present.

We found that CXCL12 was constitutively expressed in the GCL, IPL, and OPL of the mouse retina (Figure 7A). However, its role in the normal retina is not known. Since it is chemotactic for CXCR4 positive immune cells such as T cells and monocytes, it might be actively involved in intraocular inflammation. CXCR4 has been implicated in ocular leukocyte trafficking in an ovalbumin-induced acute uveitis model in DO11.10 mice with CD4+ T cells genetically engineered to react with ovalbumin (22). In addition, it might promote endothelial cell migration and enhance angiogenesis, the latter effect being supported by reports that CXCL12 promotes angiogenesis in the diabetic retina in rats (23) and oxygen-induced retinopathy in mice (24).

Most adoptively transferred disease-inducing T cells require “licensing for pathogenicity” in the lung and other organs in order to induce disease in target organs (23, 25)—“a hub-and-spoke pattern” (24). Both “licensing for pathogenicity” and HMGB1 production might take place during the induction of the effector phase of EAU. Release of intraocular HMGB1 and CXCL12 on day 1 after cell transfer shown in our previous study (8) might attract “licensed” pathogenic T cells to the eye, and the later HMGB1 increase might be related to the involvement of these T cells and the damage they invoke in the retina. AMD3100 treatment might block the migration of licensed pathogenic T cells to the eye, leading to T cell apoptosis due to lack of reactivation in the target organ. Moreover, as shown in this study, AMD3100 treatment reduced the subsequent intraocular cascading and retina damage (Figures 5 and 7), resulting in a reduced frequency of naïve T cells activated by uveitogenic antigens released from the damaged retina, as demonstrated by low proliferative and cytokine production responses *in vitro* by IRBP-specific T cells from AMD3100-treated mice (Figure 6). Results in Figures 6C,D showed that AMD3100-treated APCs could not present antigens to activate T cells from controlled mice, indicating that low responsiveness of specific T cells is partially due to the reduced antigen-presenting functions of APCs such as monocytes/macrophages. Blockade of CXCR4 on APCs probably reduces the expression of positive costimulatory molecules, which is an interesting observation and needs to be further investigated.

**Figure 8:**
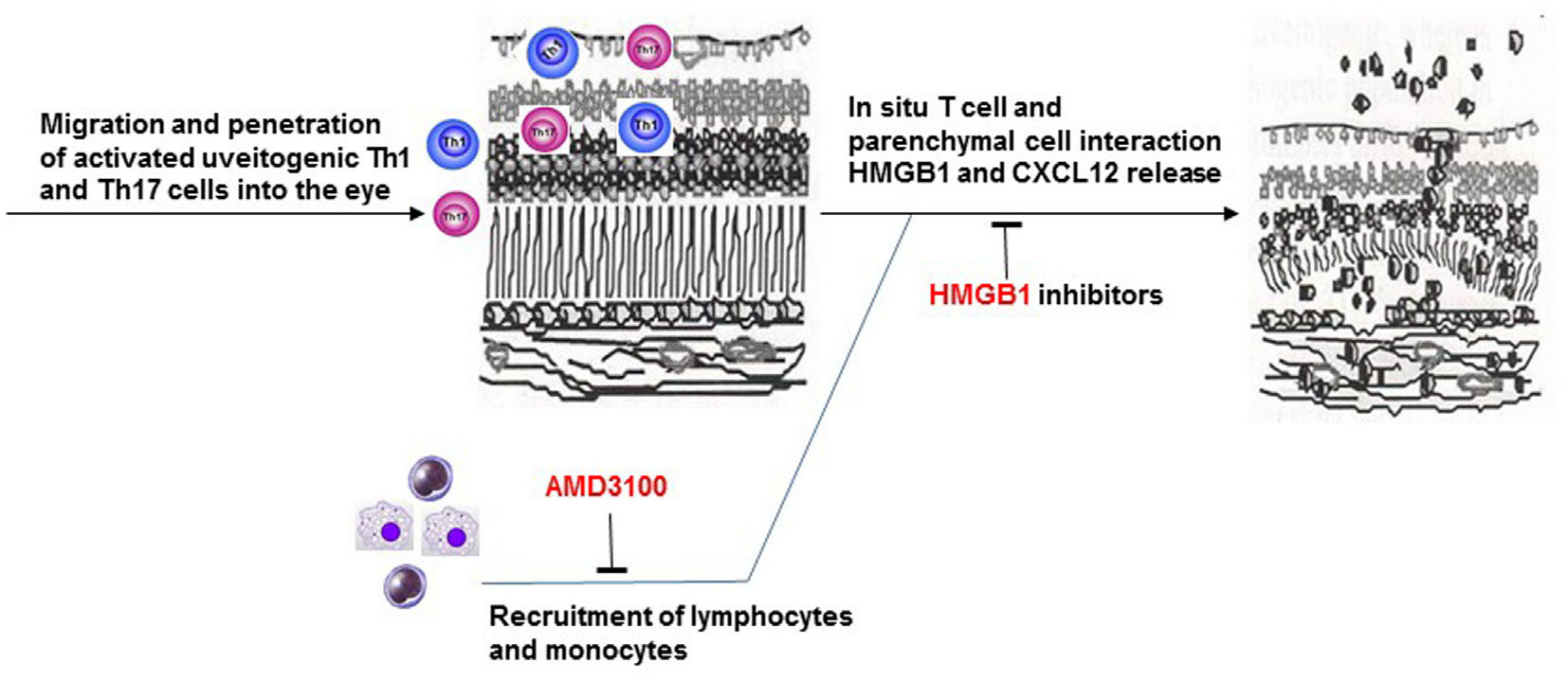
**Brief summary of the mechanism of HMGB1-CXCL12 in the pathogenesis of effector phase of EAU**.

In summary, our study demonstrates synergistic roles of HMGB1 and CXCL12 in the infiltration of inflammatory cells at a very early stage of intraocular inflammation induced by uveitogenic autoreactive T cells. In our animal model, early blockade of released HMGB1 decreases CXCL12-induced leukocyte migration into the eye and subsequent intraocular inflammation and photoreceptor cell damage (Figure 8).

## Ethics Statement

This study was carried out in accordance with the recommendations of University of Louisville. Institutional approval was obtained and institutional guidelines regarding animal experimentation were followed.

## Author Contributions

GJ and JY performed the experiments and generated and analyzed the data; YW and TX assisted with the experiments and the data analysis; YZ, DS, and HK helped with the design of the experiments and edited the manuscript; and HS directed the study, planned experiments, interpreted the data, and wrote the manuscript. All authors read and approved the final manuscript.

## Conflict of Interest Statement

The authors declare that the research was conducted in the absence of any commercial or financial relationships that could be construed as a potential conflict of interest.

## Abbreviations

EAU, experimental autoimmune uveitis; HMGB1, high mobility group box 1; IRBP, interphotoreceptor retinoid-binding protein; RAGE, receptor for advanced glycation end products; tEAU, activated IRBP-specific T cell-induced EAU, TREM-1, triggering receptor expressed on myeloid cells-1.
